# Editorial: Risky behaviors faced by youth in an internet-based learning environment

**DOI:** 10.3389/fpsyg.2024.1420357

**Published:** 2024-06-06

**Authors:** Chiao Ling Huang, Seokmin Kang, Shu Ching Yang

**Affiliations:** ^1^Interdisciplinary Program of Education, Center for Teacher Education, National Chi Nan University, Nantou County, Taiwan; ^2^Department of Teaching and Learning, College of Education & P-16 Integration, The University of Texas Rio Grande Valley, Edinburg, TX, United States; ^3^Institute of Education, National Sun Yat-sen University, Kaohsiung, Taiwan

**Keywords:** risk behaviors, internet, learning environment, youth, internet-based learning, prevention program

In recent decades, there has been a notable trend toward using the internet and technology for education. During adolescence, students encounter numerous challenges and temptations that may be exacerbated by being exposed to misinformation or disinformation, social media or gaming activities. Although the positive impacts of an internet-based learning environment on students have been demonstrated (Poccoli et al., 2001; Chou and Liu, 2005; El-Sabagh, 2021), this editorial aims to address the concerns that internet-based environments may have on students and their relation to their learning based on empirical studies and seeks support systems for the next generations.

These internet-based social environments negatively impact students' cognitive functioning—especially information processing—including cognitive biases (Britt et al., 2019) and impaired reasoning and thinking skills (Bago et al., 2020; Axelsson et al., 2021). The biased cognitive system even negatively affects behaviors (Bastick, 2021), such as the use of unauthorized resources or pirated software (Bonadio et al., 2021), cyberloafing, and cyberbullying (Huang et al., 2023).

These phenomena worsened throughout the pandemic (Fu et al.; Malik et al., 2023). For example, Malik et al. examined academic cheating behaviors and perceived online effectiveness on academic performance during the COVID-19 pandemic among schools, colleges, and university students in Pakistan. Their findings revealed that a significant portion of students engaged in cheating during online exams, with 60% admitting to cheating most of the time and 30% admitting to cheating at least once during such exams. Multivariate regression analysis indicated that cheating contributed to a 26% increase in students' academic performance. The results suggest that students perceive online learning as effective, as it allows for easy attainment of academic grades. Therefore, technology-based virtual activities moderate risky behaviors.

Since millennials and Gen Z were born with technology but their parents are not ready to fully guide them to a society with an unprecedented learning environment, the gap in perception between students and their parents makes it difficult to create supportive learning environments for younger generations. This phenomenon is well described by Luo et al.. Luo et al. conducted a survey of 629 parents in Taiwan to investigate their perspectives on their young children's utilization of information communication technology (ICT), including usage patterns and the interplay with socioeconomic status. The results revealed a notable disparity. While approximately 50% of parents deemed 6 years old or older as an appropriate age for children to commence using ICT, more than 80% of children had already begun interacting with ICT before reaching that age.

This finding highlights a substantial gap between parental expectations and the actual onset of ICT engagement among children. Additionally, the parents showed a wide range of spectra, from balanced and optimistic views to value emphasis, conservatism, and negative doubts, of children's use of information communication technology rather than a monotonous binary perspective, such as being positive or negative. The results imply the intricate and multifaceted nature of parental viewpoints, which encompass both positive and negative attitudes toward children's use of ICT. Since initial learning behaviors while interacting with the world strongly influence subsequent behaviors or are at least fixed as a habit, earlier intervention by primary caregivers is needed to better control any risky behavior in internet-based learning environments.

Risky behaviors not only hinder student learning but also have impacts on individuals and their peers, including social deterioration (Hoff et al., 2012), interpersonal distress (Copeland et al., 2007; Chen et al., 2020), emotional harm (Hessler and Katz, 2010; Weiss et al., 2015), and victimization (Windle, 1994). Creating a supportive and secure learning environment has always been a crucial educational goal in offline and online settings as well. Therefore, it is essential to understand the dynamics of how risky behaviors are manifested in internet-based learning environments and how schools and educators can prevent and address such behaviors among students.

The present Research Topic calls readers' attention to youths' risky behaviors in an online context, and empirical studies on this topic have investigated the perspectives of caregivers (Luo et al.) and students (Malik et al.). During the pandemic, internet-based learning environments for students became more influential. Future research is recommended to elucidate the neural mechanism of risky behaviors, the challenges faced by adolescents and parents and their interactions, the remedies that help overcome these challenges, and the design and effectiveness of intervention programs.

## Author contributions

CH: Conceptualization, Writing – review & editing. SK: Writing – original draft. SY: Conceptualization, Writing – review & editing.
